# 20-Hydroxyecdysone Regulates the Expression of 30 Genes Specifically Expressed in Larval Digestive Tube of the Silkworm, *Bombyx mori*

**DOI:** 10.3390/insects16030291

**Published:** 2025-03-11

**Authors:** Jiahao Li, Mulin Xia, Songyao Huang, Guangxie Zhang, Yuncheng Tang, Pingzhen Xu, Meirong Zhang

**Affiliations:** 1 Jiangsu Key Laboratory of Sericultural and Animal Biotechnology, School of Biotechnology, Jiangsu University of Science and Technology, Zhenjiang 212100, China; 221111802128@stu.just.edu.cn (J.L.); 221211801108@stu.just.edu.cn (M.X.); 231211802122@stu.just.edu.cn (S.H.); 241111802127@stu.just.edu.cn (G.Z.); tyc@meetsoho.net (Y.T.); xpz198249@just.edu.cn (P.X.); 2 Key Laboratory of Silkworm and Mulberry Genetic Improvement, Ministry of Agriculture and Rural Affairs, Sericultural Scientific Research Center, Chinese Academy of Agricultural Sciences, Zhenjiang 212100, China; 3 Jiangsu SOHO Silkworm Seed Company, Nanjing 210012, China

**Keywords:** *Bombyx mori*, digestive tube, 20-hydroxyecdysone, larval-to-larval molting, wandering

## Abstract

Nutrients needed to fuel the processes of growth, development, and reproduction, and to form the proteins in the cocoon, are derived from the larval stage. The amount of leaf ingested in the fifth instar silkworm larvae accounted for about 85% of the whole instars. Nutrients were absorbed following digestion in the digestive tube. We previously reported the successful identification of 227 silkworm larval digestive juice proteins, wherein most of the genes of the identified proteins had high expression features in the midgut, and 30 of them were midgut-specific genes. We chose 30 genes and detected their expression characteristics in the digestive tube, and investigated the developmental expression profiles in the midgut of silkworm larvae from the day-3 fourth instar to the wandering period, as well as the expression dynamics after the 20-hydroxyecdysone (20E) treatment. The genes were specifically expressed in the larval digestive tube. The expression of *BMSK0013805* was activated, and that of the other 29 genes were suppressed by 20E. The ecdysteroid ingestion dramatically suppressed the protease activity in the larval midgut, which revealed that 20E may also directly affect digestive activity. The present study is the first to demonstrate that the expression control mechanism of these genes is primarily hormonal regulation, which contributes to furthering our understanding of the mechanism of the genes expressed in the larval digestive tube during development.

## Simple Summary

In this study, we chose 30 genes that were specifically expressed and the encoding digestive juice proteins in the digestive tube to identify and detect their expression characteristics in the digestive tube. Our data demonstrated that the expression of these genes was primarily regulated by hormones. The ecdysteroid ingestion dramatically suppressed gene expression and protease activity in the larval midgut. Overall, our findings contribute to furthering the understanding of the expression control mechanism of the genes expressed in the larval digestive tube during development.

## 1. Introduction

The silkworm, *Bombyx mori*, a model insect of Lepidoptera, has been extensively studied in both basic and applied research, not only for its economic significance but also to advance our understanding of the general biological mechanisms shared by insects [1,2]. The silkworm is a complete metamorphosis insect that goes through the following four stages: egg, larval, pupal, and adult. However, feeding occurs only during the larval stage of the silkworm’s life cycle, so the larvae must digest sufficient nutrients to support the normal development of the remaining three stages. As a result, during the larval period, the body size and weight of the silkworm increase significantly, corresponding to the progression of its developmental stages. For example, the weight of the terminal fifth instar larvae is approximately 10,000 times that of a newly hatched silkworm larva [3]. Silkworm larvae need to go through five instars to reach the pupal stage, with feeding only interrupted at the end of each instar when they enter the molting state.

Mulberry leaves are the sole source of nutrition for silkworms, so the digestive tube of silkworm larvae is particularly developed and well-adapted to efficiently digest mulberry leaves and absorb nutrients. The digestive tube runs longitudinally along the central body cavity from the mouth to the anus in larval stages. It can be divided into three parts, the foregut, midgut, and hindgut, and this structural differentiation appears to maximize the digestive efficiency [3,4]. During the larval period, most of the physiological activities, including digestive activity, are affected or controlled by two main hormones, the ecdysone and juvenile hormone (JH), and the levels of these hormones in the hemolymph are significantly influenced by nutritional conditions [5,6]. Interestingly, the maintenance of the nutritional condition is closely linked to the digestive activity. Therefore, the hormonal regulation of digestive enzyme activity likely operates in response to the nutritional conditions, ensuring that optimal nutritional status is maintained for normal larval growth and development [7]. Additionally, digestive activity is also regulated by nervous, paracrine, and prandial mechanisms [8]. The larval growth rate and developmental process are directly regulated by the nutritional condition and are indirectly regulated via hormonal and growth factors [9,10,11].

Under malnourished conditions, the intrinsic titer controls of ecdysone and JH are disrupted, preventing normal development [12]. In tobacco hornworm, *Manduca sexta*, the JH titer increases when larvae are subjected to starvation conditions that block the pupal differentiation of the wing and leg imaginal disks [5,9]. In silkworm, the fifth instar day-0 larvae are fed with ecdysone, and ecdysteroid ingestion suppresses carbohydrate hydrolysis in the midgut [13]. During the silkworm larval–larval molting and intermolt feeding period, the control mechanism of carbohydrate hydrolysis sequentially changes from dietary to hormonal regulatory, according to the developmental process, and the digestive system is essentially under the dual regulation of nutrients and ecdysone [14]. The dietary carbohydrates’ hydrolyzing activity is regulated by ecdysone, wherein an increase in the ecdysone titer decreases that activity during silkworm larval–pupal metamorphosis [15].

Among the three compartments—the foregut, midgut, and hindgut—the midgut is the most important, accounting for about 78% of the total length of the digestive tube. Its primary functions are digestion and absorption, facilitated by the action of enzymes in digestive juice [3]. In addition, the genes encoding enzymes involved in digestive activity are typically tissue-specific, being expressed only in the midgut. For example, on day 3 of the fifth instar silkworm larvae, 216 of the 5588 genes expressed in the midgut display tissue specificity [2]. We previously reported that a total of 227 silkworm larval digestive juice proteins had been successfully identified, wherein most of the genes had high expression features in the midgut. Of these, 30 genes were primarily identified as midgut-specific genes based on the microarray data from the SilkDB database [3]. Food intake in silkworms increases significantly between each instar. Before reaching the next instar, a slight rise in the ecdysteroid titer occurs to initiate the molting process, and feeding will resume once the molting is complete [16]. Throughout the latter half of the fifth instar, stepwise increases in ecdysteroid titer are observed, and the titer starts to increase gradually and elevates steeply to form a peak during larval–pupal metamorphosis, whereas the last instar larvae molt to the pupa and never resume feeding [16]. In the latter half of the fifth instar, silkworms start to massively synthesize silk proteins in the silk gland, and the silk gland grows rapidly [17]. So far, investigations into the larval digestion mechanisms have been limited to the regulation of dietary carbohydrates’ hydrolyzing activities and protease activity and secretion; little work has focused on the expression pattern of genes specifically expressed in the digestive tube of insects, despite their importance in nutrient production. Therefore, in the present study, we chose the 30 genes that were specifically expressed in the digestive tube and the encoding digestive juice proteins to identify and detect their expression characteristics in the digestive tube. We also aimed to determine the developmental expression profile of each gene and the trypsin enzyme activity in the midgut of silkworm larvae from day 3 of the fourth instar to the wandering period and after treatment with 20-hydroxyecdysone (20E).

## 2. Materials and Methods

### 2.1. Experimental Animals and 20-Hydroxyecdysone Treatment

Larvae of the *B*. *mori* strain “radiation seven” were reared on fresh mulberry leaves at a stable temperature of 25 °C. The fourth instar larvae entered the molting state on day 5, and the duration of the fifth instar stage was 8 days. In the terminal fifth instar stage, the larvae stopped feeding and started spinning to make cocoons, and the silkworms proceeded with larval–pupal metamorphosis after the completion of silk spinning.

The 20-hydroxyecdysone (20E), an ecdysteroid (A506554), was purchased from Sangon Biotech Co., Ltd. (Shanghai, China), and was dissolved in anhydrous ethanol and diluted to 40 mg L^–1^ using sterile distilled water. Dissolved 20E or sterile distilled water was mixed with the mulberry leaves. The day-5 fifth instar larvae were fed the 20E or a control diet.

### 2.2. Identification of the 30 Genes

We used the accession numbers of SilkDB 3.0 to search in SilkDB 3.0 (https://silkdb.bioinfotoolkits.net (accessed on 6 April 2024)) [18] and obtained the chromosome position, nucleic acid and protein sequences, functional annotation, and expression profiles based on the transcriptome data of the 30 genes. The hidden Markov model in the Pfam database was used to search the protein families of the 30 genes [19].

### 2.3. Sample Preparation

The middle period of the larval–larval molt was designated as the molting stage. The larval midgut of the day-3 fourth instar, molting fourth instar, day-0 fifth instar (immediately after ecdysis, feeding 0 h), day-3 fifth instar, day-5 fifth instar, day-7 fifth instar, wandering-1 (immediately after maturing, with a few mulberry leaves in the midgut), wandering-2 (silkworm urine without mulberry leaves in the midgut), and wandering-3 (silkworm urine being excreted) was collected. The midgut was collected from the larvae of the 20E treatment group after 6 and 12 h. The foregut, midgut, and hindgut of the day-3 fifth instar larvae were collected. We dissected six to nine individuals in each experiment to investigate the expression profile of the 30 genes.

### 2.4. Reverse Transcription PCR and Reverse Transcription–Quantitative PCR Analysis

To analyze the expression patterns of the 30 genes in the foregut, midgut, and hindgut of the day-3 fifth instar larvae by reverse transcription–quantitative PCR (RT-qPCR) and reverse transcription PCR (RT-PCR), the total RNA was extracted using the TRIzol reagent (Invitrogen, Carlsbad, CA, USA). The total RNA was extracted from the larval midgut samples of the day-3 fourth instar, molting fourth instar, day-0 fifth instar, day-3 fifth instar, day-5 fifth instar, day-7 fifth instar, wandering-1, wandering-2, wandering-3, and the 20E treatment groups after 6 and 12 h. The total RNA concentrations were quantified. The first-strand complementary deoxyribonucleic acid (cDNA) was synthesized using the PrimeScript Reverse Transcriptase kit (TaKaRa, Dalian, China) according to the manufacturer’s instructions. RT-qPCR was performed as previously described, and the expression data were normalized and visualized using TBtools (Chao Chen, South China Agricultural University, Guangzhou, China, v2.142) [3]. The translation initiation factor 4a (*TIF-4A*) gene was used as an intrinsic control. The specific primers for each gene are shown in Table S1.

### 2.5. Expression Pattern Analysis of 20E Signaling Pathway

The RNA-seq data for the 20E signaling pathway genes in the silkworms (*EcR*, *USP*, *Br*, *E74A*, *E75*, *E93*, and *FTZ-F1*) were obtained from SilkDB 3.0, and the accession numbers were, respectively, BMSK0005452, BMSK0001871, BMSK0004693, BMSK0008350, BMSK0005523, BMSK0015146, and BMSK0000386. The filtered expression data were normalized and visualized using TBtools to reveal the expression profiles in the midgut and for the developmental period analysis.

### 2.6. Trypsin Activity Assay

To evaluate the trypsin enzyme activity, the midgut samples were homogenized in ice-cold water at a ratio of 1:9 (*w*/*v*), and subsequently centrifuged at 10,000× *g* for 10 min at 4 °C. The resulting supernatant from the cold midgut was used for the analysis. The enzymatic activity of trypsin was determined using the commercial kits (Solarbio, Beijing, China).

### 2.7. Statistical Analysis

Data were presented as the mean ± SEM of three independent biological replicates with three technical replicates, unless otherwise noted. All of the analyses were performed using GraphPad Prism 9 software (GraphPad Software, LLC, San Diego, CA, USA, v9.X). The significance of difference was determined by Student’s *t*-test and denoted by * *p* < 0.05, ** *p* < 0.01, *** *p* < 0.001, and **** *p* < 0.0001.

## 3. Results

### 3.1. Identification and Expression Analysis in Digestive Tube

We previously reported that a total of 227 silkworm larval digestive juice proteins were successfully identified, wherein most of the genes of the identified proteins had high expression features in the midgut, and 30 of them were midgut-specific genes based on microarray data from the day-3 fifth instar silkworm larvae [3]. The 30 genes expressed specifically in the midgut were determined based on the database containing a large amount of transcriptome data for exploring silkworm gene expression in various tissues and developmental periods in SilkDB 3.0 (Figure S1). The 30 genes were identified in the silkworm genome sequence, and the information, including accession numbers, brief descriptions, chromosomal distribution, the Pfam domain, EuKaryotic Orthologous Group (KOG), and Gene Ontology (GO) annotations, was collected for each (Table 1). They were distributed on 15 chromosomes, with chromosomes 9, 18, and 21 each containing four genes (Table 1). The functional analysis of the Pfam domain, and KOG and GO annotations, revealed that they had the main functions of digesting dietary proteins, carbohydrates, and lipids. The Pfam domains PF00089, PF00246, PF01433, PF00557, PF00450, and PF01321, and the KOG annotations KOG2650, KOG1046, and KOG2413, were involved in protein digestion and absorption (Table 1 and Table 2). The Pfam domains PF00128, PF01055, and PF01522, and the KOG annotations KOG0471 and KOG1066, were involved in carbohydrate transport and metabolism, and the Pfam domains PF00135 and PF00151, and the KOG annotation KOG1516, were involved in lipid transport and metabolism (Table 1 and Table 2). The functional annotations of the Pfam domain PF00245 and KOG annotation KOG4126 were involved in inorganic ion transport and metabolism. The transcription activator MBF2 (PF15868) plays an important role in the development of the silkworm as a tissue-specific and stage-specific coactivator via forming a complex with MBF1 and the DNA-binding regulator FTZ-F1 [20]. Gamma interferon-inducible lysosomal thiol reductase (PF03227) plays a role in the immune system. The Pfam domain and KOG and GO annotations of BMSK0012049, BMSK0012234, and BMSK0013414 were not found in the database.

**Figure 1:**
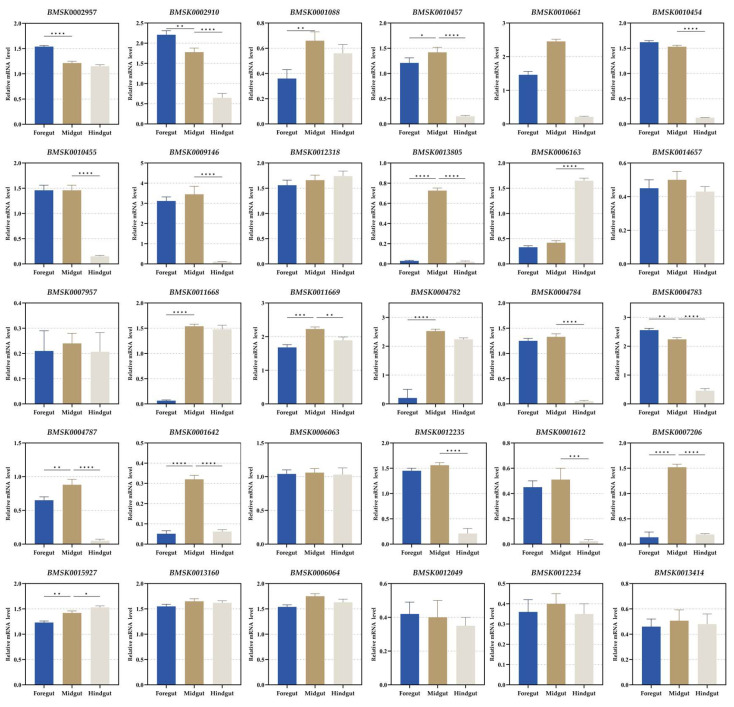
Expression pattern of each gene in the foregut, midgut, and hindgut of the day-3 fifth instar silkworm larvae. The significance of difference was determined by Student’s *t*-test and denoted by * *p* < 0.05, ** *p* < 0.01, *** *p* < 0.001, and **** *p* < 0.0001.

The digestive tube of silkworm larvae can be divided into the foregut, midgut, and hindgut according to its function and structure. There are only midgut transcriptome data in SilkDB 3.0. Day 3 of the fifth instar of the silkworm is the boundary for the whole larval development stage [2]. Thus, we investigated the expression characteristics of the 30 genes in the foregut, midgut, and hindgut on day 3 of the fifth instar larvae by RT-PCR and RT-qPCR, respectively. *BMSK0013805*, *BMSK0001642*, and *BMSK000726* were specifically expressed in the midgut (Figure 1 and Figure S2). *BMSK0002910*, *BMSK0010454*, *BMSK0009146*, *BMSK0004784*, *BMSK0004783*, *BMSK0004787*, and *BMSK0001612* were expressed in both the foregut and midgut (Figure 1 and Figure S2). *BMSK0011668* and *BMSK004782* were expressed in both the midgut and hindgut (Figure 1 and Figure S2). The other 18 genes were expressed in the foregut, midgut, and hindgut (Figure 1 and Figure S2). All of the 30 genes were expressed in the midgut (Figure 1 and Figure S2). Thus, we still took the midgut as the target organ for the following study.

### 3.2. Developmental Expression Profile of Each Gene from Day 3 of Fourth Instar to Wandering Period

**Figure 2:**
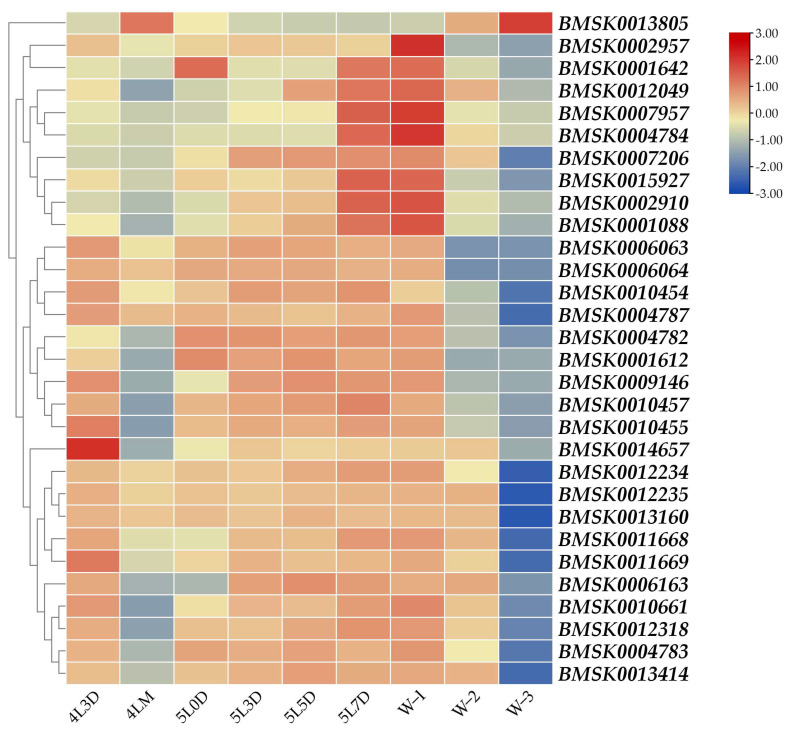
Heatmap of expression profiles of each gene in the midgut of silkworm larvae from the day-3 fourth instar to the wandering period. 4L3D: day-3 fourth instar; 4LM: fourth larval molting; 5L0D: day-0 fifth instar; 5L3D: day-3 fifth instar; 5L5D: day-5 fifth instar; 5L7D: day-7 fifth instar; W-1: wandering-1; W-2: wandering-2; W-3: wandering-3.

A small rise in the ecdysteroid titer occurs before the molting of the fourth instar; then, following larval–larval molting, the titer decreases rapidly and becomes undetectable, and the titer then starts to increase gradually and elevates steeply from the wandering period to the spinning period [16]. We examined the developmental expression profile of each gene in the midgut of the silkworm larvae from the day-3 fourth instar to the wandering period. Particularly, *BMSK0013805* exhibited a unique expression pattern, with significantly higher expression during the molting fourth instar (4LM) and wandering-3 (W-3) stages, when the ecdysteroid titers were elevated (Figure 2). In contrast, the expression levels of the other 29 genes decreased during these stages (Figure 2). Additionally, with the exception of *BMSK0013805*, the remaining 29 genes maintained high expression levels from day 0 to day 7 of the fifth instar, particularly after the gluttonous stage (Figure 2). These results suggested that all 30 genes were likely regulated by ecdysteroid, with 29 of them being suppressed, while *BMSK0013805* was positively regulated.

### 3.3. Regulation of Expression of Each Gene by 20E Treatment

**Figure 3:**
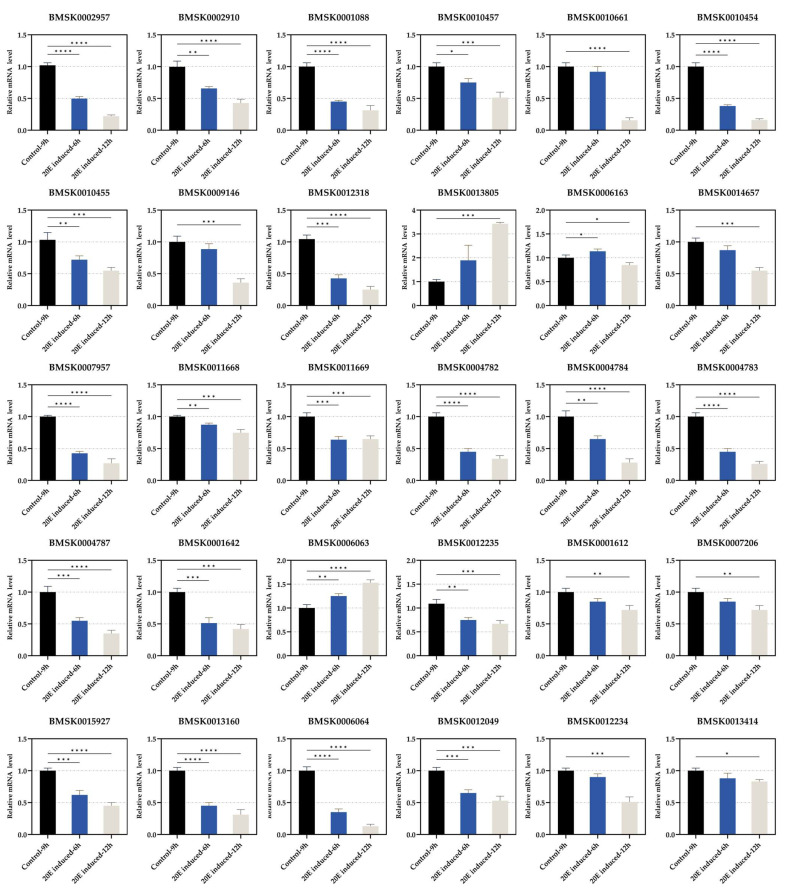
The expression status of each gene in the midgut after the 20-hydroxyecdysone (20E) treatment. The experiments were repeated three times. The significance of difference was determined by Student’s *t*-test and denoted by * *p* < 0.05, ** *p* < 0.01, *** *p* < 0.001, and **** *p* < 0.0001.

It was suggested in Figure 2 that the expression levels of the 30 genes in the midgut were affected by ecdysteroid. We therefore examined the gene expression profiles of the 20E treatment after 6 and 12 h. The expression level of *BMSK0013805* was significantly increased with the 20E treatment (Figure 3). Meanwhile, the expression level of *BMSK0013805* was increased stepwise from the wandering-1 period to the wandering-3 period, and was higher in the molting fourth instar and relatively lower from day 0 to day 7 of the fifth instar (Figure 2). These results suggest that the expression of *BMSK0013805* was activated by ecdysteroid. In contrast, the expressions of the other 29 genes were suppressed by ecdysteroid (Figure 3).

### 3.4. The Potential Regulatory Mechanism of Each Gene by 20E in Midgut

**Figure 4:**
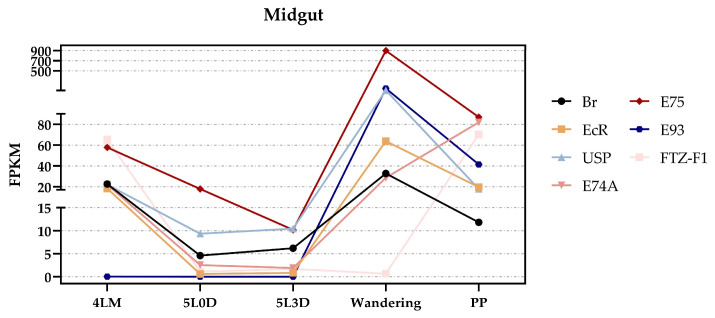
Transcriptomic expression data of 20E signaling pathway genes in the midgut from the SilkDB 3.0 database, including *EcR*, *USP*, *Br*, *E74A*, *E75*, *E93*, and *FTZ-F1*, from the fourth molting instar to the wandering period. 4LM: fourth larval molting; 5L0D: day-0 fifth instar; 5L3D: day-3 fifth instar; PP: pre-pupa.

Since we discovered that the expression levels of these 30 genes were altered by the 20E treatment, we wondered whether this regulation mechanism was direct or indirect. The 20-hydroxyecdysone (20E) signaling pathway, which regulates many biological processes in insects, is mediated by a heterodimer complex consisting of the Ecdysone Receptor (EcR) and Ultraspiracle (USP). This EcR-USP complex then directly activates a set of primary response genes, including *Broad Complex* (*Br*), *E74A*, *E75*, *E93*, and *FTZ-F1*. Therefore, to further investigate the 20E regulatory mechanism, we extracted the expression data of these genes in the midgut from the SilkDB 3.0 database (Figure 4). Interestingly, except for *E74A, E93*, and *FTZ-F1*, high expression levels were observed during both the molting fourth instar and wandering stages (same for the wandering-3 stage), which precisely correspond to the suppressed or increased expression of the 30 genes (Figure 2). Based on these results, we concluded that the altered expression of the 30 genes may be directly regulated by the 20E signaling pathway. However, compared to similar studies in insects like *Drosophila melanogaster* and *Aedes aegypti* [21,22], where RNAi is commonly used to determine which genes in the 20E downstream signaling pathway are directly involved, the RNAi efficiency in silkworm larvae is limited. This limitation made it challenging to fully elucidate the regulatory mechanism of these 30 genes. Therefore, more specific and efficient genetic tools need to be developed in future studies to further validate these findings.

### 3.5. Trypsin Activity from Day 3 of the Fourth Instar to the Wandering Period and Its Regulation by 20E Treatment in Midgut

**Figure 5:**
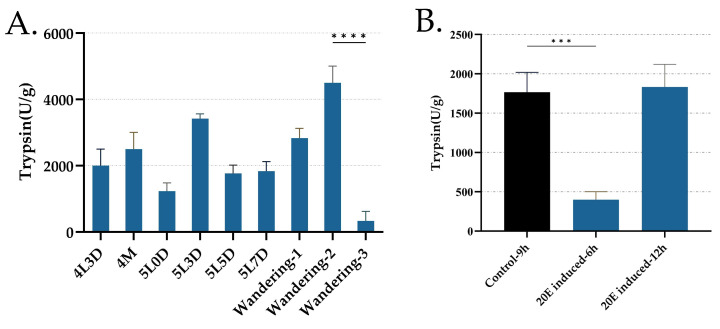
Trypsin enzyme activity. (**A**) The trypsin enzyme activity in the midgut silkworm larvae from the day-3 fourth instar to the wandering period. (**B**) The trypsin enzyme activity between the control and 20-hydroxyecdysone (20E) treatment groups. The significance of difference was determined by Student’s *t*-test and denoted by, *** *p* < 0.001, and **** *p* < 0.0001.

There was an interesting phenomenon that caught our attention. After treating the day-5 fifth instar larvae with 20E for 6 h, the silkworms exhibited several characteristics typical of the wandering stage. For instance, the larvae frequently raised their heads, secreted small amounts of silk, and, most notably, their bodies became slightly translucent. However, these characteristics disappeared soon after they consumed fresh mulberry leaves two hours later. Therefore, we speculated that the 20E may not only affect the transcriptional level of digestive genes but also transiently and directly affect the digestion process. According to the functional annotation, most of the 30 genes were associated with protein digestion, so we chose trypsin, an important protease in the midgut, as a representative enzyme for the assessment to further examine whether or how the digestion process was affected. Interestingly, although the results of the trypsin activity share similar traits with the expression pattern of the 30 genes, such as a sudden decline in the wandering-3 stage, several differences were observed. Notably, while the expression levels of most of the 30 genes peaked in the wandering-1 stage, the trypsin activity reached its peak in the wandering-2 stage (Figure 5A). Additionally, the trypsin activity recovered quickly after the 20E treatment compared to the expression levels of the 30 genes (Figure 3 and Figure 5B), aligning with our observation that feeding can resume. These results indicate that, while 20E can influence the digestion process, its effects are not immediate. Furthermore, the rapid recovery of the trypsin activity suggests that some other mechanisms can possibly mitigate the effects of the 20E treatment, allowing silkworms to resume feeding and successfully enter the molting stage. However, more research is necessary to confirm and elucidate these mechanisms.

## 4. Discussion

Only the larvae feed during the silkworm’s whole life cycle. The nutrients needed to fuel the processes of growth, development, and reproduction, and the proteins formed in the cocoon, are derived from the larval stage. The core value of the larval stage is feeding, absorption, and the accumulation of nutrients. The amount of leaf ingested in the fifth instar silkworm larvae accounted for about 85% of the whole instars. Nutrients are absorbed following digestion in the digestive tube. Previously, we successfully identified a total of 227 silkworm larval digestive juice proteins, wherein most of the genes of the identified proteins had high expression features in the midgut, and 30 of them were midgut-specific genes [3]. In the present study, we examined the developmental expression profile of each gene in the midgut of silkworm larvae from the day-3 fourth instar to the wandering period, and the expression dynamics after the 20E treatment. The expression of *BMSK0013805* was activated by ecdysteroid. The expressions of the other 29 genes were suppressed by ecdysteroid. Interestingly, the genes involved in the 20E signaling cascade, such as *EcR*, *USP*, *Br*, and *E75*, also exhibited high expression when the expression levels of the 30 genes changed, which suggests that the expression of these genes may directly regulated by 20E. However, there were several limitations that can be addressed in future studies to further strengthen this conclusion. For instance, incorporating a restricted-feeding control group that is not treated with 20E would help to eliminate the influence of dietary factors. Additionally, since RNAi is not feasible in silkworm larvae, more specific genetic tools, such as the GAL4-UAS system, could be designed to investigate which genes in the 20E signaling pathway directly regulate these 30 genes, as well as the mechanisms of their regulation. Moreover, we observed some intriguing phenomena in the silkworms after the 20E treatment. To investigate this, the trypsin activity was assayed as an indicator. The results suggest that 20E may also affect the digestive activity, but in a transient and reversible manner, and further research is needed to elucidate the details of this mechanism.

The functional annotation of the 30 genes showed that the genes were mainly involved in the digestion. Many of the 30 genes were also expressed in the foregut and hindgut, which were essentially determined by the structure and function of the digestive tube. The foregut does not play an important role in nutrient absorption because it is covered by a cuticle that is as impermeable as the outer surface of the insect body [23,24]. However, the foregut is an important site for mechanical and chemical digestion, and the initial carbohydrate digestion can take place in the foregut [7,24,25,26]. The midgut represents the most permeable section of the digestive tube and comprises the main site for digestion and absorption, where the cuticle lining is absent [7,23,24]. Despite being covered by a cuticle, the hindgut is responsible for the absorption of important substances before being eliminated as feces [7,23,24].

Many genes related to metabolism, proteolysis, and transport are downregulated in the midgut during the molting stage of the fourth instar; additionally, the feeding larvae of the fifth instars injected with 20E that entered a molting-like stage and displayed changes in gene expression exhibited the same patterns as observed in the actual molting state [27]. In this study, we showed that the expression levels of these genes were lower or undetectable in the molting state, wandering state, and after the 20E treatment. These results indicate that the ecdysteroid suppresses the expression of the genes in the silkworm larval midgut. The midgut tissue of insects is capable of converting ecdysone to 20E [28,29]. The hemolymph ecdysteroid titer increases at the end of the penultimate larval instar and induces the cessation of feeding behavior [16,30]. The penultimate instar larvae molt to the last instar larval stage and resume feeding. In fact, ecdysteroid is scarcely detected in the hemolymph during the feeding period [16,30]; this low level of ecdysteroid allows these genes to be activated and expressed during the intermolt feeding period. During larval–pupal metamorphosis, the larvae molt to the pupa and never resume feeding, the hemolymph ecdysteroid titer starts to increase gradually and then elevates steeply [16,30], and this increase suppresses the expression of these genes. Overall, except for *BMSK0013805*, the expression levels of the other 29 genes were significantly declined during the molting and wandering states (with a higher level of hemolymph ecdysteroid titer); in contrast, they maintained high expression levels during the feeding period (with the ecdysteroid titer scarcely detected in the hemolymph).

In the insect digestive tube, serine proteases, aminopeptidases, and carboxypeptidases are all responsible for protein digestion. Notably, serine proteases—such as trypsins, chymotrypsins, and elastases—account for 95% of the digestive activity [24,31]. Day 3 of the fifth instar marks a turning point in the silkworm’s entire larval development stage; afterwards, it enters the gluttonous stage [2,3]. In the gluttonous stage, the amount of leaf ingested and digested by the silkworm larvae quickly increases, and large amounts of proteins from mulberry leaves are digested and absorbed [32]. This phenomenon occurs because silkworm larvae require an adequate supply of amino acids for the synthesis of silk proteins, which activates lots of proteases to facilitate this process [3,33]. Similarly, the activities of alkaline phosphatase and aminopeptidase N are maintained at the same levels in the day-4 fourth instar and day-1 fifth instar until the molting of the fourth instar. Both enzyme activities suddenly decline during the molting of the fourth instar [34]. In Cluster 1, the transcript levels of the 18 genes associated with protein digestion were markedly elevated after day 3 of the fifth instar, suggesting a close association with the onset of this process. Furthermore, these findings indicate that the expression of the genes within Cluster 1 is regulated by the nutritional state during the feeding period.

Dietary carbohydrates are hydrolyzed into monosaccharides by digestive enzymes in the gut, and, subsequently, are absorbed into the hemolymph. These monosaccharides are rapidly utilized in various metabolic pathways, including glycolysis, the pentose phosphate pathway, and the synthesis of glycogen or trehalose [7,35]. Carbohydrate digestion plays a crucial role in maintaining hemolymph sugar levels, particularly in the synthesis of trehalose. In silkworms (*Bombyx mori*), the concentration of trehalose in the hemolymph is maintained at approximately 10 mM, although this concentration decreases following the cessation of feeding during the final larval stage. This regulation of hemolymph trehalose is essential for energy homeostasis during periods of fasting [36,37]. Steroidal hormones regulate the hydrolytic activity of the dietary carbohydrates in the silkworm, and the dietary carbohydrates’ hydrolyzing activity remains high throughout the last larval period, and then decreases to negligible levels until the pupal period; additionally, ecdysteroid ingestion dramatically suppresses carbohydrate processing in the larval midgut to reduce the nutritional value of the ingested diet [13,14,15]. In Cluster 2, the three genes associated with carbohydrate digestion were similar to that of the Cluster 1 genes.

*BMSK0013805* belongs to triglyceride lipases (EC 3.1.1.3) that hydrolyze the ester linkages of triglycerides. The expression of *BMSK0013805* was activated by ecdysteroid. The expression level of *BMSK0013805* was increased stepwise from the wandering-1 to wandering-3 periods, and was higher in the molting fourth instar and relatively lower from day 0 to day 7 of the fifth instar. Ecdysteroid stimulates the proliferation and differentiation of intestinal stem cells (ISCs), as well as cell death-related processes such as apoptosis and autophagy, in a concentration-dependent manner during each molting and wandering period [34,38,39,40]. Therefore, we speculate that *BMSK0013805* may be involved in midgut cell death-related processes.

## 5. Conclusions

In conclusion, this study was the first to analyze the genes that were specifically expressed and the encoding digestive juice proteins in the digestive tube of the silkworm. This was also the first study to investigate the developmental expression profile of each gene in the midgut from the day-3 fourth instar to wandering period, and the expression status after the 20E treatment. Our data demonstrated that the expression level of these genes was primarily altered by 20E. Our data also supported that 20E may directly regulated these 30 genes through the 20E cascade signaling pathway in the larval midgut. Additionally, our findings revealed that 20E may also directly affect the digestive activity, but in a transient and reversible manner. Further research is necessary to elucidate the biological significance of hormonal regulation during the feeding period and to further clarify the regulatory mechanism of ecdysteroids in silkworm feeding.

## Figures and Tables

**Table 1:** Characteristics of the 30 genes that are specifically expressed and the encoding digestive juice proteins in the larval digestive tube of the silkworm *B*. *mori*.

SilkDB 3.0 ID	SilkDB 2.0 ID	Description	Location (Chr.)	Pfam	KOG	GO
BMSK0002957	BGIBMGA003604	trypsin, alkaline C	5	PF00089	KOG3627	0005615; 0004252
BMSK0002910	BGIBMGA003568	trypsin, alkaline A	5	PF00089	KOG3627	0005615; 0004252
BMSK0001088	BGIBMGA007377	collagenase-like	3	PF00089	KOG3627	0004175; 0004252; 0006508
BMSK0010457	BGIBMGA008281	ovochymase-2-like	18	PF00089	KOG3627	0005615; 0004252
BMSK0010661	BGIBMGA008514	transmembrane protease serine 9-like	18	PF00089	KOG3627	0005615; 0004252; 0030574
BMSK0010454	BGIBMGA008279	ovochymase-2-like	18	PF00089	KOG3627	0005615; 0004252
BMSK0010455	BGIBMGA008280	chymotrypsin-2	18	PF00089	KOG3627	0005615; 0004252
BMSK0009146	BGIBMGA012791	transmembrane protease serine 9	16	PF00089	KOG3627	0005576; 0004252; 0030574
BMSK0012318	BGIBMGA007183	kallikrein-1	21	PF00089	-	-
BMSK0014657	BGIBMGA004830	carboxypeptidase B	25	PF00246	KOG2650	0005576; 0004181; 0008270
BMSK0007957	BGIBMGA009477	zinc carboxypeptidase	14	PF00246	KOG2650	0005576; 0004181; 0008270
BMSK0004782	BGIBMGA008060	aminopeptidase N-1	9	PF01433	KOG1046	0031225; 0005886; 0004177
BMSK0004784	BGIBMGA008062	membrane alanyl aminopeptidase-like	9	PF01433	KOG1046	0031225; 0005886; 0004177; 0008237; 0008270
BMSK0004783	BGIBMGA008061	aminopeptidase N-3	9	PF01433	KOG1046	0031225; 0005886; 0004177; 0008237; 0008270
BMSK0004787	BGIBMGA008017	aminopeptidase N-7	9	PF01433	KOG1046	0031225; 0005886; 0004177; 0008237; 0008270
BMSK0006063	BGIBMGA001640	metallopeptidase family M24	11	PF00557	KOG2413	0005829; 0046872; 0070006; 0006508
BMSK0007206	BGIBMGA010349	serine carboxypeptidase-like 51	12	PF00450	-	0005794; 0016021; 0004185; 0006915; 0030448; 0075307
BMSK0006064	BGIBMGA001639	creatinase	11	PF01321	KOG2413	0005829; 0046872; 0070006; 0006508
BMSK0001642	BGIBMGA006066	maltase A1	4	PF00128	KOG0471	0004558; 0032450
BMSK0012235	BGIBMGA001569	sucrase-isomaltase, intestinal-like	21	PF01055	KOG1066	0005783; 0031160; 0090599; 0030246; 0016052
BMSK0015927	BGIBMGA013757	polysaccharide deacetylase	28	PF01522	-	-
BMSK0013805	BGIBMGA008141	alpha-esterase 49 precursor	24	PF00135	KOG1516	80030
BMSK0006163	BGIBMGA011895	pancreatic triacylglycerol lipase	11	PF00151	-	0005576; 0046872; 0016042
BMSK0001612	BGIBMGA008818	alkaline phosphatase	3	PF00245	KOG4126	0031225; 0005886; 0004035; 0046872
BMSK0011668	BGIBMGA004286	transcription activator MBF2	20	PF15868	-	-
BMSK0011669	BGIBMGA004285	ACN81326.1, Bm122	20	PF15868	-	-
BMSK0013160	BGIBMGA011074	gamma interferon-inducible lysosomal thiol reductase	23	PF03227	-	-
BMSK0012049	BGIBMGA002366	uncharacterized protein LOC101742057	21	-	-	-
BMSK0012234	BGIBMGA001568	lysosomal alpha-glucosidase-like	21	-	-	-
BMSK0013414	BGIBMGA011598	uncharacterized protein OBRU01 20501, partial	23	-	-	-

Note: “-” means that no Pfam, KOG, and GO accession number was found.

**Table 2:** The characteristics of the expression of the genes involved in digestion.

Cluster	Functional Annotation	No. of Genes	Pfam	Name of Genes
1	Protein digestion	18	PF00089	BMSK0002957, BMSK0002910, BMSK0001088, BMSK0010457, BMSK0010661, BMSK0010454, BMSK0010455, BMSK0009146, BMSK0012318
			PF01433	BMSK0004782, BMSK0004783, BMSK0004784, BMSK0004787
			PF00246	BMSK0014657, BMSK0007957
			PF00557	BMSK0006063
			PF00450	BMSK0007206
			PF01321	BMSK0006064
2	Carbohydrate digestion	3	PF00128	BMSK0001642
			PF01055	BMSK0012235
			PF01522	BMSK0015927
3	Lipid digestion	2	PF00135	BMSK0013805
			PF00151	BMSK0006163

## Data Availability

Data are contained within the article and Supplementary Materials.

## Supplementary Materials

The following supporting information can be downloaded at: https://www.mdpi.com/article/10.3390/insects16030291/s1, Figure S1. Tissues and developmental period expression profile of each gene. The expression values of each gene in various tissues during various growth stages (instar, larval, wandering, pupal, and moth) with different colors. Figure S2. Expression pattern of each gene in the foregut, midgut, and hindgut of the day-3 fifth instar silkworm larvae. Reverse transcription polymerase chain reaction (RT-PCR) was performed, and the *BmTIF-4A* gene was used as the internal control. Table S1. Primers of genes for reverse transcription polymerase chain reaction (RT-PCR) and RT–quantitative PCR (RT-qPCR).
