# Supplementary figures and images for: 20-Hydroxyecdysone Regulates the Expression of 30 Genes Specifically Expressed in Larval Digestive Tube of the Silkworm, Bombyx mori
Source: Insects. 2025 Mar 11;16(3):291. doi: 10.3390/insects16030291 (PMC11942947; doi:10.3390/insects16030291)

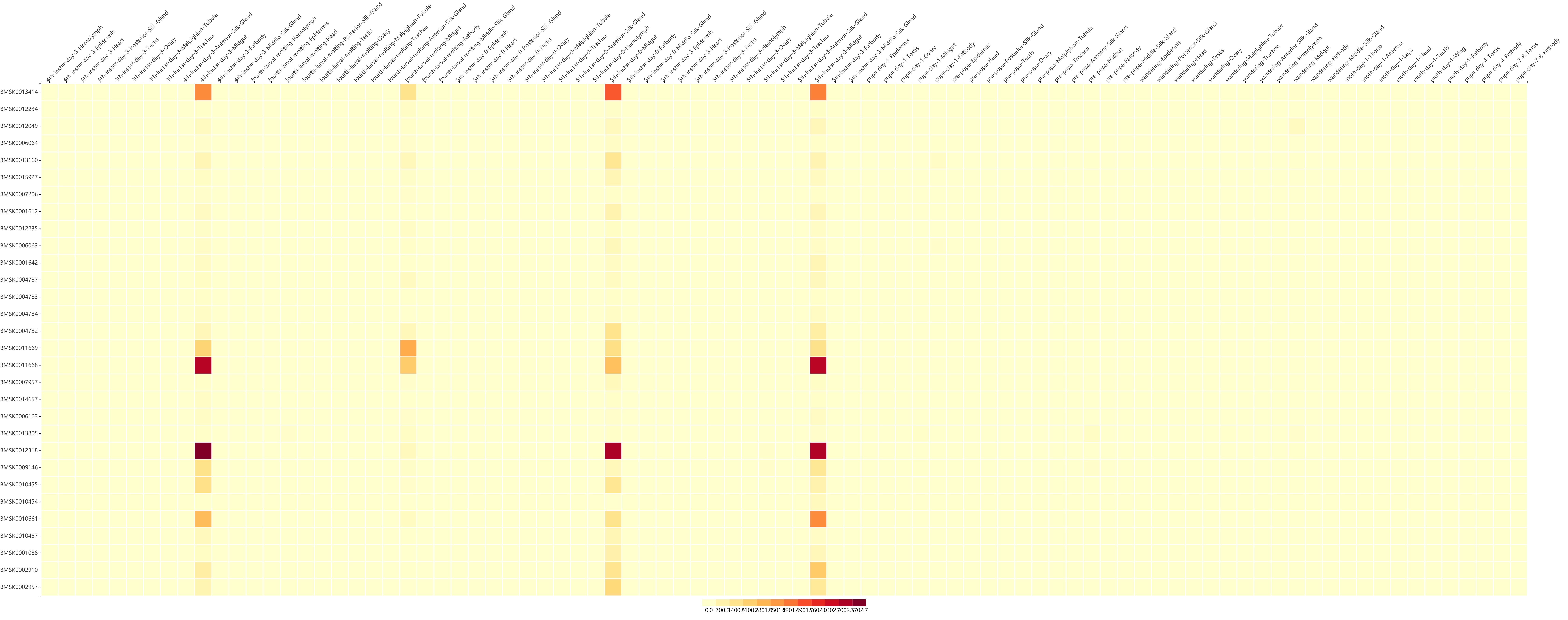

Supplement: Supplementary file 1 [file insects-16-00291-s001.zip › insects-3453420-supplementary/Figure S1.jpg]

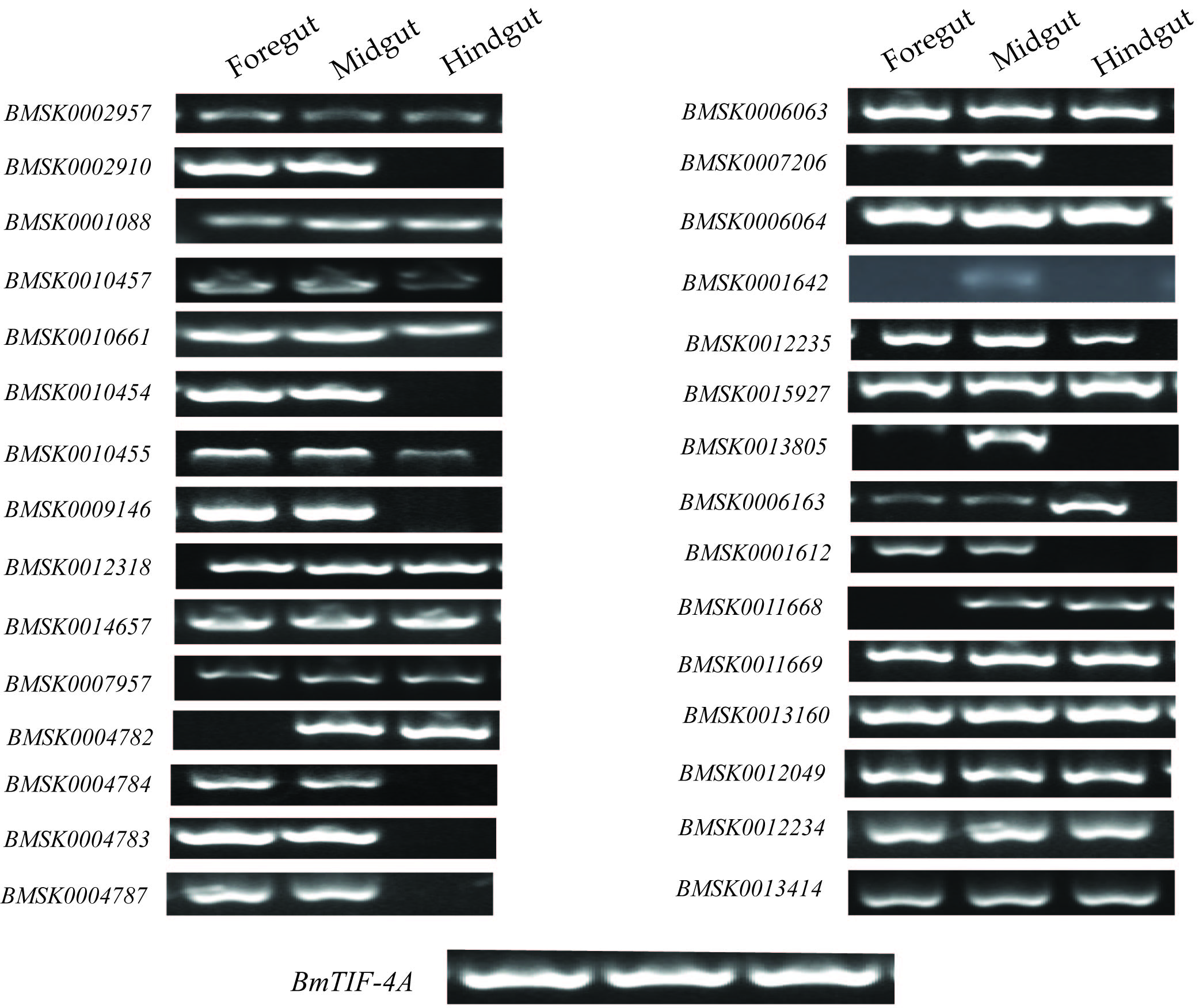

Supplement: Supplementary file 1 [file insects-16-00291-s001.zip › insects-3453420-supplementary/Figure S2.jpg]
